# Vein Transillumination Versus the Standard Method in Neonatal Venous Cannulation: A Randomized Trial

**DOI:** 10.7759/cureus.90891

**Published:** 2025-08-24

**Authors:** Zurina Zainudin, Melissa Anne Nunis, Zi Han Lim

**Affiliations:** 1 Department of Pediatrics, Faculty of Medicine and Health Sciences, Universiti Putra Malaysia, Selangor, MYS

**Keywords:** neonate, nicu, success rate, vein transillumination device, venous cannulation

## Abstract

Introduction: Peripheral venous cannulation is an essential procedure in neonatal intensive care units (NICUs) but is often associated with high failure rates. Near-infrared vein visualization devices may enhance vein identification and reduce the number of attempts; however, evidence in neonatal populations remains limited. This study aimed to compare the first-attempt success rates of peripheral venous cannulation using a vein transillumination device versus the standard method in neonates with prior punctures. Secondary analyses explored patient- and performer-related factors associated with successful cannulation.

Methodology: This study conducted a prospective, randomized controlled trial from October 2022 to January 2024 in the NICU at Hospital Sultan Abdul Aziz Shah, Universiti Putra Malaysia. Only neonates of ≥ 35 weeks of gestation requiring venous access with at least one prior venipuncture were enrolled. The participants were randomized into standard (control) and AccuVein AV500-assisted (intervention) cannulation groups. All procedures were performed by 14 trained medical officers. First-attempt success was documented as the primary outcome, while secondary outcomes included associations with patient, performer, and procedural factors.

Results: A total of 120 neonates were enrolled (60 per group). The overall first-attempt peripheral venous cannulation success rate was 47 (39.2%). No significant differences were observed between the control (21, 35%) and the intervention (26, 43.3%) groups (*P* > 0.05). Vein palpability was notably associated with cannulation success (odds ratio (OR) 7.86, 95% confidence interval (CI): 2.22-27.85, *P* = 0.001). Right-sided cannulation was also linked to improved cannulation success (OR 3.44; 95% CI: 1.15-10.32, *P* = 0.028). Conversely, four or more previous punctures reduced the likelihood of successful peripheral venous cannulation (OR 0.30; 95% CI: 0.09-0.99, *P* = 0.048).

Conclusions: Vein transillumination did not significantly improve the first-attempt peripheral venous cannulation success rate. However, vein palpability, fewer prior punctures, and right-sided cannulation were associated with superior outcomes.

## Introduction

Peripheral venous cannulation is a common procedure in neonatal intensive care units (NICUs), providing essential access for fluids, medications, blood products, parenteral nutrition, and neonatal resuscitation [1,2]. Nonetheless, achieving successful cannulation in neonates, particularly those requiring repeated punctures, remains a significant challenge. Factors such as small vessel size, difficulty in palpating veins, and reduced vein visibility contribute to higher failure rates compared to adults [3], with up to one-third of cases unsuccessful on the first attempts [4]. Multiple attempts cause pain and distress, increase the risk of complications such as hematoma, phlebitis, infection, and nerve injury [5], and can lead to frustration among healthcare providers [6].

Various techniques, including active warming, ultrasonography, and transillumination, have been used to improve success rates [7-10], but their effectiveness in neonates is limited due to technical challenges and the small size of neonatal veins. Near-infrared vein visualization offers real-time imaging of superficial veins and was originally developed for military use before being adapted for neonatal care to reduce cannulation attempts and improve success rates [1,11]. However, evidence in the pediatric population is mixed [12]. Some studies report improved outcomes, while others show no significant benefit [13-15]. For example, Inal and Demir [3] conducted a randomized controlled trial in pediatric patients aged 0 to 3 years and reported higher success with the AccuVein AV400 compared to standard methods, whereas Conversano et al. [14], in a pseudo-randomized controlled trial involving patients aged 0 to 18 years, observed comparable outcomes between the intervention and control groups. Although most research has been conducted in broader pediatric populations, few studies specifically evaluate neonates, particularly preterm infants [1,11,16].

Exploring strategies that enhance peripheral venous cannulation success is critical, considering the notable failure rates of first attempts in neonates and the risks associated with multiple attempts. Near-infrared vein visualization devices have shown potential for improving vein identification and reducing the number of attempts and complications. Nevertheless, their effectiveness in neonates remains inadequately investigated. Consequently, evaluating the effects of this technology can provide valuable insights into its role in optimizing neonatal vascular access, reducing procedural distress, and improving resource utilization in NICUs.

This study sought to address the research question: Does the use of a vein transillumination device improve the first-attempt success rate of peripheral venous cannulation compared to the standard method in neonates with at least one prior puncture? We hypothesized that the device would increase the likelihood of first-attempt success. As a secondary, exploratory objective, we examined patient-related and performer-related characteristics that may be associated with successful cannulation.

## Materials and methods

This prospective, randomized controlled trial was conducted in the NICU of Hospital Sultan Abdul Aziz Shah (HSAAS), Universiti Putra Malaysia (UPM), from October 2022 to January 2024. HSAAS is a tertiary referral center serving approximately 570,000 residents in the central region of Malaysia.

Neonates with a gestational age of 35 weeks or more who required peripheral venous cannulation for intravenous antibiotics or fluids, and had at least one prior skin puncture (from either cannulation or blood sampling), were eligible for inclusion. Cannulation was performed at the discretion of the attending medical team as part of routine care. Exclusion criteria included significant hemodynamic instability, the need for immediate resuscitation, and the presence of dysmorphic features (e.g., trisomy 21) or congenital limb anomalies. Participant selection was conducted using a convenience sampling method. 

**Figure 1:**
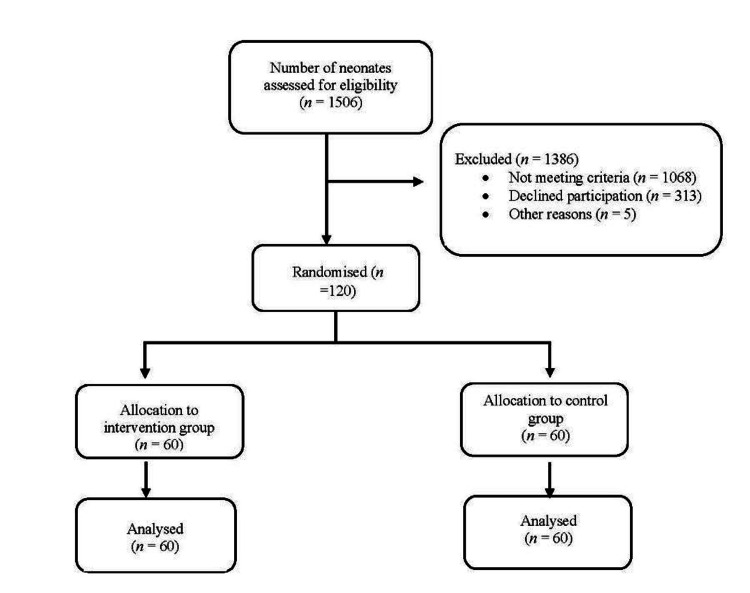
Flowchart of recruitment and randomization into control and intervention groups. Image credit: Created by the authors.

The required sample size for this study was calculated using G*Power (version 3.1.9.7) [17]. Power analysis indicated that 124 newborns (62 in each group) were needed to detect a statistically significant difference with an α error of 0.05 and 95% power, accounting for a 5% non-response rate. Eligible participants were randomly assigned at the time venous cannulation was required to either the control group, which received standard cannulation, or the intervention group, which underwent cannulation using the vein transillumination device (Figure 1). Group allocation was concealed from medical officers until the point of randomization and remained fixed thereafter to maintain blinding. Parents were also not informed of group assignments.

Block randomization was employed to ensure balanced group sizes. The randomization sequence was generated using the web-based Sealed Envelope^TM^ system (Sealed Envelope Ltd., London, UK), with a 1:1 allocation ratio, incorporating stratification by skin tone based on the Fitzpatrick scale: light skin tones (I-III) and dark skin tones (IV-VI). The allocation sequence was prepared by an independent officer from the Clinical Research Center (CRC) who was not involved in the trial. Neonates were enrolled by volunteer medical officers, and group assignments followed the pre-generated block randomization list. No modifications were made to the pre-specified trial outcomes after study initiation.

In the control group, cannulation was performed according to routine NICU practice without the use of assistive vein visualization devices. The performer inspected the neonate's hands and feet to assess skin color, vein visibility and palpability, and the presence of prior puncture wounds, bruising, hematomas, or swelling. This was followed by standard aseptic preparation before cannulation. In the intervention group, the same preliminary inspection and assessment were performed. Vein selection was then guided using the Accuvein AV500 vein transillumination device, which projects a near-infrared image of superficial veins onto the skin surface. Once a suitable vein was identified, the skin was prepared using standard aseptic technique before cannulation. 

In both groups, cannulation was performed by pediatric medical officers with at least six months of NICU experience, using 24-gauge intravenous cannulas (B. Braun, Hessen, Germany). A successful attempt was defined as the successful insertion of the cannula into the vein with free blood flow and subsequent flushing with normal saline without signs of infiltration or extravasation. A maximum of three attempts was permitted per participant, with each attempt defined as a single skin puncture. If cannulation was unsuccessful after three attempts, the procedure was deemed unsuccessful. In such cases, another medical officer not involved in the study performed the cannulation to ensure continuity of standard care. For these subsequent attempts, the decision to use the AccuVein AV500 was left to the attending medical officer's discretion, regardless of the participant’s original group allocation.

**Figure 2:**
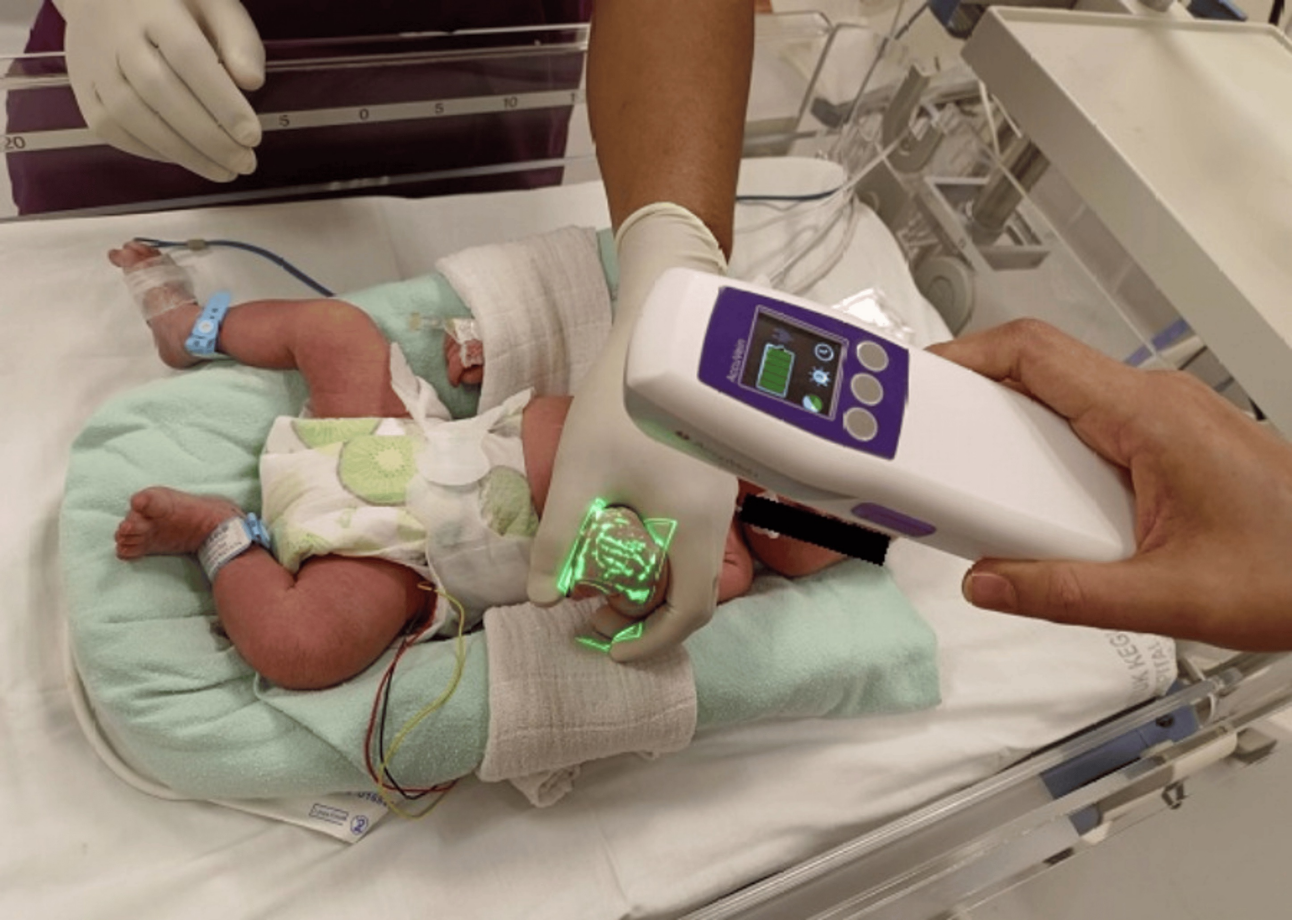
AccuVein AV500 used for locating veins in neonates. Image credit: Photograph taken by the authors with the guardian's consent.

The AccuVein AV500 is a non-invasive, handheld vascular imaging system that uses infrared and red lasers to safely visualize subcutaneous blood vessels up to 10 mm deep without contacting the patient’s skin (Figure 2). The device is lightweight, produces no heat or radiation, and eliminates the risk of burns or infection transmission. It provides highly accurate vein mapping to assist in cannulation. In this study, the lead investigator conducted a one-hour structured training session on the use of the AccuVein AV500, covering proper handling, positioning, and techniques for optimal vein visualization. Following the training, each officer was allotted one week of hands-on practice with the device. Cannulation skills were evaluated to ensure standardization in patient preparation and the cannulation procedure. No significant changes to the study methods, including eligibility criteria, were made after trial commencement.

The primary outcome was the first-attempt cannulation success rate, while secondary outcomes included factors associated with successful and unsuccessful attempts. Data were analyzed using SPSS version 29.0 (IBM Corp., Armonk, NY). Descriptive statistics summarized demographic and clinical characteristics. The chi-square test or Fisher's exact test was used to compare categorical variables, while continuous variables were compared using the independent t-test or Mann-Whitney U test as appropriate. The association between potential predictors and successful cannulation was assessed using simple logistic regression, with a *P*-value <0.05 considered statistically significant.

The study received approved from the Ethics Committee for Research Involving Human Subjects, UPM (JKEUPM-2021-039), and was registered with the National Medical Research Registry (Trial registration: NMRR-21-1259-59378) and the CRC at HSAAS, UPM. Written informed consent was obtained from the parents or legal guardians of all participants before enrolment.

## Results

Baseline characteristics

During the study period from October 2022 and January 2024, 125 neonates were enrolled. Two neonates were inadvertently recruited twice, and three cases had missing data without an identifiable cause. Following these exclusion, 120 neonates (60 in each group) were included in the final analysis. Based on Table 1, the baseline characteristics recorded by both groups were comparable and demonstrated no significant differences. The trial was also completed according to schedule, without early termination or stopping.

**Table 1:** Baseline characteristics of the study population (n = 120). ^a^Independent t-tests. ^b^Chi-square tests.

Variable	Control (*n* = 60)		Intervention (*n* = 60)		*t* or *χ*^2^	*P*-value
	*n* (%)	Mean (SD)	*n* (%)	Mean (SD)		
Age (in weeks)						
1st week (*n* = 75)	35 (58.3)		40 (66.7)		3.68	0.298^b^
2nd week (*n* = 15)	9 (15)		6 (10)			
3rd week (*n* = 11)	8 (13.3)		3 (5)			
≥4th week (*n* = 19)	8 (13.3)		11 (18.3)			
Gender						
Female	29 (48.3)		22 (36.7)		1.671	0.196^b^
Male	31 (51.7)		38 (63.3)			
Gestational status						
Pre-term	17 (28.3)		17 (28.3)		0.001	0.58^ b^
Full term	43 (71.7)		43 (71.7)			
Birth weight (gram)		2796.8 (733.94)		2711.5 (688.12)	0.657	0.512^ a^
Maternal comorbidities						
Yes	52 (86.7)		44 (73.3)			
No	8 (13.3)		16 (26.7)			
Skin tone						
I to III (Light)	48 (80)		49 (81.7)		0.455	0.068^b^
IV to VI (Dark)	12 (20)		11(18.3)			
Vein characteristics					0.054	0.817^ b^
Visibility (yes)	50 (83.3)		47 (78.3)		0.484	0.487^ b^
Palpability (yes)	36 (60)		28 (46.7)		2.143	0.143^ b^
Presence of bruises/ hematoma/ swelling					1.345	0.246^ b^
Yes	46 (76.7)		51 (85)			
No	14 (23.3)		9 (15)			
Number of previous punctures						
1	10 (16.7)		7 (11.7)		1.512	0.680^b^
2-3	30 (50)		36 (60)			
4 and more	20 (33.3)		17 (28.4)			

Most neonates involved in this study were in their first week of life, males, and Malay. The predominantly full-term participants also documented a 39.2-week mean gestational age and approximately 2.7 kg mean birth weight. Furthermore, a majority of the neonates had light skin tone (Fitzpatrick types I-III). Over 75% of the participants were also observed with vein visibility, while half had palpable veins. Moreover, most neonates demonstrated evidence of bruises, hematomas, or swelling, with prior punctures being common. Regarding maternal characteristics, 96 (80%) of the mothers involved in this study had comorbidities, including pregnancy-induced hypertension, gestational diabetes and anemia in pregnancy.

Most of the 14 medical officers from the Pediatric Department of HSAAS who volunteered to participate in this study were senior medical officers with over two years of experience. On average, the officers possessed between 3 and 6.5 years of experience in general and pediatric care, including training in the NICU. Weekly, the medical officers performed an average of seven intravenous cannulations. Based on self-assessment data, half of the officers considered their cannulation skills as average, while the rest rated their competency as moderately high. In this study, the same group of medical officers conducted peripheral venous cannulation in the control and intervention groups to minimize performer-related factor influences on the success rate of the procedure.

Success rate of peripheral venous cannulation

The overall first-attempt success rate for peripheral venous cannulation was 47 (39.2%) of 120 attempts. Success rates did not differ significantly between the intervention and control groups (21, 35%, versus 26, 43.3%; *P* > 0.05). Successful cannulation was more frequently achieved on the right side (29, 61.7%), compared to the left side (18, 38.3%). Additionally, the upper limb demonstrated a higher success rate (40, 85.1%) compared to the lower limbs (7, 14.9%). No harms or unintended effects were reported in either group.

Association between patient characteristics and successful peripheral venous

This study employed simple logistic regression analysis to examine the effect of individual patient characteristics on the likelihood of first-attempt peripheral venous cannulation success. As shown in Table 2, the presence of palpable veins was strongly associated with a higher success rate, with an odds ratio (OR) of 7.86 (95% CI: 2.22-27.85, *P* = 0.001). In contrast, neonates who had undergone four or more previous punctures were significantly less likely to achieve successful cannulation on the first attempt (OR 0.30; 95% CI: 0.09-0.99; *P* = 0.048). Notably, within the intervention group, these characteristics did not demonstrate a statistically significant association with first-attempt success.

**Table 2:** Association between patient characteristics and successful peripheral venous cannulation in the control group (n = 120). Note: Statistical analysis = simple logistic regression analysis. *Reference group. ^e^Unable to compute. OR, odds ratio; CI, confidence interval

Variable	Control		Intervention	
	OR (95% CI)	P	OR (95% CI)	P
Age (in weeks)				
1st week	*	*	*	*
2nd week	2.12 (0.48-9.32)	0.322	0.47 (0.05-4.43)	0.507
3rd week	1.69 (0.36-7.94)	0.505	4.67 (0.39-55.51)	0.226
4th week and above	1.69 (0.36-7.94)	0.505	2.80 (0.71-10.98)	0.14
Gender				
Female	0.65 (0.23-1.82)	0.415	2.05 (0.69-6.11)	0.2
Male	1.53 (0.55-4.29)	0.415	0.50 (0.16-1.46)	0.2
Gestational status				
Pre-term	1.24 (0.40-3.82)	0.714	1.45 (0.46-4.61)	0.529
Full term	0.81 (0.26-2.50)	0.714	0.69 (0.22-2.19)	0.529
Birth weight (gram)	1.00 (0.99-1.01)	0.89	1.00 (0.99-1.01)	0.973
Maternal comorbidities (none)				
Yes	*	*	*	*
No	0.15 (0.02-1.34)	0.091	0.33 (0.08-1.34)	0.122
Skin tone				
I to III	1.09 (0.30-3.92)	0.896	0.58 (0.15-2.20)	0.424
IV to VI	0.92 (0.26-3.31)	0.896	1.72 (0.46-6.49)	0.424
Vein characteristics				
Visibility (yes)	e	e	2.07 (0.50-8.54)	0.315
Palpability (yes)	7.86 (2.22-27.85)	0.001	1.42 (0.49-4.13)	0.516
Presence of hematoma/bruises/swelling (no)	2.07 (0.62-6.98)	0.238	2.73 (0.65-11.56)	0.171
Number of previous punctures				
1	2.25 (0.56-9.00)	0.251	2.82 (0.57-14.04)	0.205
2 to 3	1.73 (0.62-4.85)	0.299	0.62 (0.21-1.81)	0.378
4 or more	0.30 (0.09-0.99)	0.048	1.02 (0.31-3.30)	0.976

Association between performer characteristics and successful peripheral venous

The duration of training as pediatric officers and medical officers was notably associated with successful peripheral venous cannulation, with increases of 1.29- and 1.28-fold, respectively (*P* < 0.10) (Table 3). Conversely, no medical officer characteristic showed a significant association with cannulation success in the intervention group. Table 4 presents the relationship between cannulation site and successful cannulation in both groups. In the control group, right-sided cannulation was 3.44 times more likely to succeed compared to the left side (OR = 3.44; 95% CI: 1.15-10.32; *P* =0.028). A similar trend was observed in the intervention group.

**Table 3:** Association between performer characteristics and successful peripheral venous cannulation in the control versus intervention group. *Omit ratings of 1, 2, and 5 as they hold no value, i.e., value 0. Statistical analysis = simple logistic regression analysis. OR, odds ratio; CI, confidence interval

Variable	Control		Intervention	
	OR (95 % CI)	P	OR (95 % CI)	P
Duration of training as medical officer	1.29 (0.98-1.69)	0.07	1.16 (0.92-1.47)	0.213
Duration of pediatric training	1.28 (0.98-1.68)	0.067	1.02 (0.75-1.38)	0.901
Duration of NICU training	1.19 (0.90-1.57)	0.232	1.00 (0.68-1.46)	0.999
Number of IV access per week	1.02 (0.83-1.25)	0.87	1.04 (0.92-1.17)	0.553
Self-rated skill*				
3	0.48 (0.14-1.62)	0.238	2.67 (0.66-10.80)	0.169
4	2.07 (0.62-6.97)	0.238	0.38 (0.09-1.52)	0.169

**Table 4:** Association between the cannulation site and successful peripheral venous cannulation in the control versus intervention group (n = 120). Statistical analysis = simple logistic regression analysis. OR, odds ratio; CI, confidence interval

Variable	Control		Intervention	
	OR (95% CI)	P	OR (95% CI)	P
Side of cannulation				
Right	3.44 (1.15-10.32)	0.028	0.86 (0.30-2.50)	0.787
Left	0.29 (0.10-0.87)	0.028	1.16 (0.40-3.35)	0.787
Location of cannulation				
Upper limb	1.98 (0.53-7.34)	0.307	1.31 (0.30-5.71)	0.717
Lower limb	0.51 (0.14-1.87)	0.307	0.76 (0.18-3.32)	0.717
Cannulation at the site with pre-existing bruises/hematoma or swelling (Yes)	0.44 (0.15-1.30)	0.138	0.27 (0.07-1.06)	0.061

## Discussion

The overall first-attempt success rate for peripheral venous cannulation in this study was 39.2%, which is considerably lower than the success rates reported in other studies, where rates of up to 62.3% have been observed in neonates [18,19]. The lower rate is likely due to differences in the study population. Unlike most previous studies, which generally included neonates undergoing cannulation for the first time, this study exclusively involved both preterm and term neonates with prior skin punctures. These repeated attempts may have resulted in bruising, scarring, or vein depletion, thereby reducing vein visibility and accessibility, and ultimately lowering the likelihood of successful cannulation. 

Comparisons between the first-attempt success rates observed in the control and intervention groups indicated no notable variations (21, 35%, vs. 26, 43.3%; *P* > 0.05). Similar outcomes regardless of vein visualization device use were reported by Çağlar et al. [1] and Raut et al. [16]. Meanwhile, Phipps et al. noted an improving success trend for peripherally inserted central catheters (PICC) with similar devices (86% vs. 75%, *P* = 0.08). The variability in efficacy across studies suggests that device performance may be context dependent [11]. Furthermore, the data suggest that challenges inherent to neonatal cannulation, such as small and fragile veins, compounded by prior puncture-related damage, may limit the benefits of vein visualization devices even in the hands of experienced operators. 

In this study, palpable veins were strongly associated with higher first-attempt success rates for peripheral venous cannulation (OR 7.86, 95% CI: 2.22-27.85; *P* = 0.001), consistent with the findings of Al-Awaisi et al. [20]. This association was significant in the control group only, which may be explained by operators' reliance on traditional assessment methods, such as palpation and visual cues, where veins that were easier to feel were more likely to be successfully cannulated. In the intervention group, the vein visualization device likely guided cannulation to the most suitable veins regardless of palpability, potentially masking the effect of palpable veins. However, it is important to note that the observation of vein accessibility and successful cannulation was present across both groups, with a possible explanation being that vein accessibility was preserved despite prior attempts, and the skill of the operators helped overcome the anticipated difficulties in cannulation.

Higher first-attempt success rates were also observed for right-sided cannulation (OR 3.44, 95% CI: 1.15-10.32; *P* < 0.05). This effect was significant only in the control group, with no similar association in the intervention group. The reasons for this discrepancy remain unclear, and further investigation is warranted to determine whether anatomical, technical, or operator-related factors contribute to this trend. Additionally, a higher number of prior skin punctures was associated with reduced cannulation success rates (OR 0.30, 95% CI: 0.09-0.99; *P* < 0.05), a finding supported by previous studies reporting that repeated attempts can damage veins and hinder successful cannulation [21,22].

Although no significant association was observed between medical officers’ characteristics and first-attempt success rates, years of experience showed an almost notable trend, reflecting the relative uniformity of skill among officers. This study focused on pediatric medical officers as primary cannulation performers, whereas previous studies often involved pediatric nurses [13,20,23] or anesthesiologists [24,25], which may influence the effect of operator experience.

In the intervention group, no significant association was found between patient or performer characteristics, or the site of cannulation, and successful peripheral venous cannulation. The vein visualization device did not increase overall first-attempt success, but it was used in all the cases in the control group after three failed attempts. Among users, 11 (81.2%) found it useful and expressed willingness to use it again. The device may be a valuable adjunct in patients with difficult intravenous access, helping operators visualize veins not easily seen by the naked eye and enhancing confidence during cannulation. Its limited impact on first-attempt success may be due to its inability to determine vein depth, requiring performers to estimate puncture depth, which can lead to through-and-through punctures or extravasation. Additionally, small, fragile neonatal veins, especially those with prior punctures, may further limit its effectiveness. Nevertheless, the device remains a useful tool in challenging cases, and its effectiveness may be maximized through thorough training and experience.

A key strength of this study is its practical, real-world design, reflecting challenges commonly encountered in routine NICU practice. The randomized controlled trial design, combined with the inclusion of neonates who had undergone prior punctures, allowed a controlled evaluation of the vein transillumination device in a clinically relevant and underrepresented population. The use of experienced pediatric medical officers to perform cannulation further enhances the practical applicability of the findings, providing valuable insights into factors influencing cannulation success in routine NICU settings.

This study had several limitations warranting consideration. The relatively small sample size and single-center design restricted the generalizability of the findings to wider settings, particularly institutions with elevated admission rates or different patient populations. Furthermore, this study could not blind the intervention, possibly introducing performance bias risk, as operators were aware of the group assignments despite standardized protocols. No objective competency threshold was established before commencing this study. Consequently, AccuVein AV500 proficiency variations were unavoidable, although training sessions were provided. Variability in device familiarity might also influence the observed efficacy. Previous studies suggested that enhanced operator experience can improve success rates [4,20]. The technical limitation of the device, particularly in accurately assessing vein depth in neonates, requires the operator to estimate puncture depth, which may contribute to lower success rates. Moreover, the potential advantages of vein visualization technology may be less apparent when highly experienced medical officers perform the cannulation, as their skills alone could achieve high success rates.

## Conclusions

This study provides valuable insights into factors influencing the success of peripheral venous cannulation in neonates. The findings suggest that the use of a vein transillumination device may not offer significant additional benefits over the standard technique, though the conclusion should be interpreted cautiously in view of the modest sample size, marginal power shortfall, and potential variability in operator experience. The first-attempt success rate may also not be substantially improved with the device, particularly as the study involved neonates with prior repeated cannulation attempts rather than in first-time cases. 

In contrast, vein palpability, the side of puncture, and the number of previous punctures emerged as key predictors of successful cannulation. Although vein visualization devices can facilitate vein mapping, their effectiveness may be influenced by vein condition and operator proficiency. Future research incorporating standardized proficiency assessments, accurate vein depth evaluation, and enhanced training protocols will be important to better define the role of vein visualization devices in improving cannulation success among neonatal populations.
